# NUCB1 Suppresses Growth and Shows Additive Effects With Gemcitabine in Pancreatic Ductal Adenocarcinoma via the Unfolded Protein Response

**DOI:** 10.3389/fcell.2021.641836

**Published:** 2021-03-29

**Authors:** Yong-Qiang Hua, Ke Zhang, Jie Sheng, Zhou-Yu Ning, Ye Li, Wei-dong Shi, Lu-Ming Liu

**Affiliations:** ^1^Minimally Invasive Treatment Center, Fudan University Shanghai Cancer Center, Shanghai, China; ^2^Department of Oncology, Shanghai Medical College, Fudan University, Shanghai, China

**Keywords:** pancreatic ductal adenocarcinoma, NUCB1, unfolded protein response, autophagy, m^6^A modification

## Abstract

Pancreatic ductal adenocarcinoma (PDAC) is a highly aggressive cancer with poor patient prognosis. A cellular stress response mechanism called the unfolded protein response (UPR) has been implicated in PDAC progression. More recently, nucleobindin 1 (NUCB1), a calcium-binding protein, has been shown to control the UPR but its precise role in PDAC has not been explored. Here, we found that downregulation of NUCB1 was associated with poor prognosis in patients with PDAC. Functionally, NUCB1 overexpression suppressed pancreatic cancer cell proliferation and showed additive effects with gemcitabine (GEM) *in vitro* and *in vivo*. Moreover, by controlling ATF6 activity, NUCB1 overexpression suppressed GEM-induced UPR and autophagy. Last but not least, we uncovered METTL3-mediated m^6^A modification on NUCB1 5′UTR via the reader YTHDF2 as a mechanism for NUCB1 downregulation in PDAC. Taken together, our study revealed crucial functions of NUCB1 in suppressing proliferation and enhancing the effects of gemcitabine in pancreatic cancer cells and identified METTL3-mediated m^6^A modification as a mechanism for NUCB1 downregulation in PDAC.

## Introduction

Pancreatic ductal adenocarcinoma (PDAC) is responsible for more than 90% of all pancreatic diseases and is a highly devastating cancer with poor clinical prognosis (Kleeff et al., 2016; Orth et al., 2019). PDAC is the most fatal cancer with <10% of 5-year survival (Siegel et al., 2020). Several risk factors are associated with PDAC, including obesity and type-2 diabetes (Calle et al., 2003; Rahn et al., 2018), as well as lifestyle habits such as smoking and alcohol consumption (Blot et al., 1988). In addition, mutations in *BRCA1, TP53*, or *CDKN2A* have also been reported as risk factors (Pihlak et al., 2017; Hu et al., 2018).

Prognosis for PDAC patients is highly determined by disease stage at the time of diagnosis, and the lack of early detection tools presents a major challenge in improving clinical outcomes for PDAC patients. Although surgical resection followed by adjuvant chemotherapy serves as a current treatment strategy, only a very small percentage (10–20%) of PDAC patients present early-stage tumors while majority (80–90%) are late stage tumors with distant metastasis (Gillen et al., 2010; Werner et al., 2013).

Recent advances in chemotherapy have led to the introduction of gemcitabine, a nucleoside analog, as a first-line treatment for PDAC (Samanta et al., 2019), as well as 5-fluorouracil (Endo et al., 2019). Gemcitabine displays cytotoxic activity based on interference with DNA synthesis and has been shown to achieve clinical benefit and symptom improvement in 20–30% of PDAC patients (Heinemann, 2001). Unfortunately, many patients with PDAC are unable to achieve complete therapeutic benefits due to limited drug response and rapid drug resistance by tumor cells (Grasso et al., 2017). Indeed, drug resistance is a major impediment in PDAC treatment and is currently an area of major interest in the cancer field (Quinonero et al., 2019; Sarantis et al., 2020). Understanding the mechanisms that promote drug resistance is therefore critical in improving therapeutic strategies and clinical outcomes for PDAC patients.

The unfolded protein response (UPR) is an adaptive pro-survival cellular mechanism that is elicited in response to alterations in the function of the endoplasmic reticulum (ER) (Madden et al., 2019). During cancer progression, tumor cells endure tumor microenvironment and oncogenic stress, resulting in the accumulation of misfolded proteins in the ER that causes ER stress (Cubillos-Ruiz et al., 2017). Over the past decade, studies have revealed an important role for the UPR in the development and progression of breast, liver, and colon cancers (Shuda et al., 2003; Scriven et al., 2009; Jin et al., 2016). The UPR involves the activation of three ER membrane proteins, inositol-requiring transmembrane kinase/endoribonuclease 1α (IREα), PKR-like ER kinase (PERK), and activating transcription factor 6 (ATF6) (Madden et al., 2019), and ensures cell survival by regulating the transcription of genes involved in proliferation and apoptosis (Lindholm et al., 2017).

Previous studies on non-receptor guanine nucleotide exchange factors (GEFs) have indicated that these factors are able to fuel metastatic cancer progression (Barbazan et al., 2016). Interestingly, one member, nucleobindin 1 (NUCB1), has been shown to modulate the UPR *via* inhibition of ATF6 activity (Tsukumo et al., 2007). NUCB1 is a 63-kDa calcium-binding protein with multiple domains, including a leucine rich zipper, carboxypeptidase-like motifs, two zinc binding sites, and two EF-hands (Miura et al., 1992, 1996; Wendel et al., 1995; Kanuru et al., 2013). The precise physiological and biochemical functions of NUCB1, however, are not well understood and whether NUCB1 plays a role in PDAC has not been explored.

In this study, we found that downregulation of NUCB1 was associated with poor prognosis of PDAC patients. Functionally, NUCB1 overexpression suppressed proliferation and enhanced the effects of gemcitabine in pancreatic cancer cells *in vitro* and *in vivo*. Furthermore, by modulating ATF6 activity, NUCB1 overexpression blocked the effects of gemcitabine on the UPR and autophagy. Lastly, we uncovered m^6^A modification in NUCB1 5′UTR by METTL13 *via* the reader YTHDF2.

## Materials and Methods

### Bioinformatics Analysis

Gene expression data and prognostic values for CCDC88A, CDC88C, NUCB1, and NUCB2 were obtained from The Cancer Genome Atlas (TCGA) pancreatic ductal adenocarcinoma (PDAC) dataset (collectively called PAAD).

### Study Subjects

The study was performed following the protocol approved by the Institutional Ethical Review Committee of Fudan University Shanghai Cancer Center (Shanghai, China). PAAD patients (*n* = 100), who underwent surgical treatment between January 2007 and December 2008 at Fudan University Shanghai Cancer Center (Shanghai, China), were enrolled in the study after providing written, informed consents. Clinical information on patients was obtained from review of medical records for a 5-year follow-up study. Clinical stages were determined in compliance with the American Joint Committee on Cancer (AJCC) staging system. PAAD patient samples were collected during operation and kept in wax for immunohistochemical staining. Twenty-five pairs of PAAD tumor samples and adjacent non-tumorous tissue were surgically resected, and snap frozen in liquid nitrogen immediately and stored at −80°C.

### Quantitative Reverse Transcription-Polymerase Chain Reaction (qRT-PCR)

Total RNA was extracted using Trizol (Invitrogen) following manufacturer's instructions. mRNA level was quantified by qRT-PCR using SYBR^®^Green (Thermo Fisher Scientific) on ABI 7300 instrument (Applied Biosystems). GAPDH was used for normalization. All data represent average of three replicates. The primers used are listed in Supplementary Table 1.

### Immunohistochemistry (IHC)

Tissue slides were deparaffinized in xylene and rehydrated in ethanol. Antigen was retrieved with 0.01M citrate buffer (pH 6.0) using microwave for 15 min. Slides were then treated with 0.3% hydrogen peroxide for 30 min. After that, the tissue sections were incubated with 10% normal goat serum for 30 min, with NUCB1 antibody (ab154262, Abcam) overnight at 4°C, and with horseradish peroxidase (HRP)-conjugated secondary antibody for 1 h at room temperature. Immunoreactivity was detected with 3,3-diaminobenzidine (DAB) solution (Vector Laboratories) and hematoxylin was used for counterstaining. The immunoreactivity score (IRS) was graded following a previous report (Sinn et al., 2014), and IRS > 3 indicated overexpression. Staining was assessed by two independent investigators.

### Cell Culture

HEK293T, HPDE pancreatic duct epithelial cell line and human PAAD cell lines (AsPC-1, BXPC3, CFPAC1, PANC-1, and SW1990) cells were obtained from the Shanghai Cell Bank. Cells were cultured in DMEM media supplemented with 10% fetal bovine serum (FBS), 100 units/mL penicillin and 100 μg/mL streptomycin.

### Plasmid Construction and Lentiviral Production

Human NUCB1 was cloned into pLVX-puro (Clontech). Short hairpin RNA (shRNA) oligos targeting NUCB1, WTAP, METTL3, METTL14, YTHDF3, or YTHDC2 (Supplementary Table 2) were annealed and cloned into pLKO.1 vector (Addgene). Lentivirus was produced in 293T cells by transfecting cells with the plasmids along with packaging plasmids psPAX2 and pMD2G following standard protocol.

### Western Blotting (WB)

Cells were lysed with RIPA buffer supplemented with proteinase inhibitor (Beyotime). Proteins were separated by sodium dodecyl sulfate-polyacrylamide gel electrophoresis (SDS-PAGE), and transferred onto nitrocellulose membranes (Millipore). The membranes were blocked with 5% skim milk. After that, the blots were incubated with primary antibodies (Supplementary Table 3) at 4°C overnight. After washing 3× with TBST buffer, membranes were incubated with secondary antibodies conjugated with HRP (Beyotime) for 1 h at room temperature. Enhanced chemiluminescence system (ECL) (Millipore) was used to detect the signal. Bands from immunoblots were quantified using ImageJ software (http://rsb.info.nih.gov/ij/, Bethesda, MD, USA) and normalized to GAPDH.

### Cell Proliferation Assay

PAAD cell viability was measured using Cell Counting Kit-8 (Dojindo Laboratories) following manufacturer's protocol. Briefly, cells were seeded in 96-well plates at a density of 2 × 10^3^ cells per well and infected with virus. After indicated time periods, 10 μL CCK-8 solution was added to each well. The cells were then incubated for 1 h at 37°C. The number of viable cells was quantified by measuring absorbance at 450 nm using Multiskan MS plate reader (Labsystems).

### Apoptosis Assay

Cells were washed 1× with ice-cold PBS, and stained with annexin V-fluorescein isothiocyanate (FITC) apoptosis detection kit (KeyGEN Biotech). FITC staining in the cells was analyzed using a flow cytometer (BD Biosciences) to quantify apoptosis.

### Mouse Experiments

All the described animal experiments were approved by the Animal Care Committee of Fudan University Shanghai Cancer Center (Shanghai, China). Twenty four-week old male nude mice (SLRC Laboratory Animal Co, Ltd.) were randomly divided into two groups (*n* = 10 per group), and 5 × 10^6^ SW1990 cells expressing NUCB1 (oeNUCB1) or control vector (oeNC) were injected subcutaneously. Twelve days post transplantation, 10 mice in each group were randomly divided into two subgroups (*n* = 5 per group) and intraperitoneally injected with 50 mg/kg GEM or vehicle (DMSO) twice a week.

Tumor volume was measured every 3 days: volume = ½ (largest diameter) × (smallest diameter)^2^. Mice were sacrificed after 24 days, and tumors were collected and weighed. Xenografts were processed for TUNEL (TdtT-mediated DUTP nick end labeling) (Roche) staining.

### m^6^A Content Analysis

m^6^A RNA Methylation Assay Kit (Abcam, ab185912) was used to measure m^6^A content in total RNA extract.

### RNA Immunoprecipitation (RIP) Assay

RIP was performed using the Magna RIP RNA-Binding Protein Immunoprecipitation Kit (Millipore) following the manufacturer's protocol. Anti-m^6^A antibody (Synaptic Systems, 202003) or isotype control IgG antibody was conjugated with magnetic Dynabeads in RIP buffer (Magna RIP Kit, Millipore), and fragmented RNAs were immunoprecipitated with the antibody complex. The precipitated RNA was reverse transcribed and analyzed by qRT-PCR using the following primers for NUCB1: F 5′-GAGGGCATGTTCTTTCAG-3′ and R 5′-ATCAGACTCAGTCGTGGG-3′.

### mRNA Stability Measurement

PAAD cells were treated with 0.2 mM actinomycin D for 30 min, and RNAs were extracted after 0, 2, 4, and 6 h. cDNA was synthesized using SuperScript IV reverse transcriptase (Thermo Scientific) and oligo d(T) primer, and the level of mRNA expression was measured by qRT-PCR.

### Statistical Analysis

All the statistical analysis was carried out using Graphpad Prism software (version 6.0). After normal distribution was confirmed using the Shapiro-Wilk test, *p-*values were calculated using Student's *t*-test and analysis of variance (ANOVA). A *p*-value < 0.05 was considered statistically significant.

## Results

### Downregulation of NUCB1 Was Associated With Poor Prognosis in PDAC Patients

**Figure 1:**
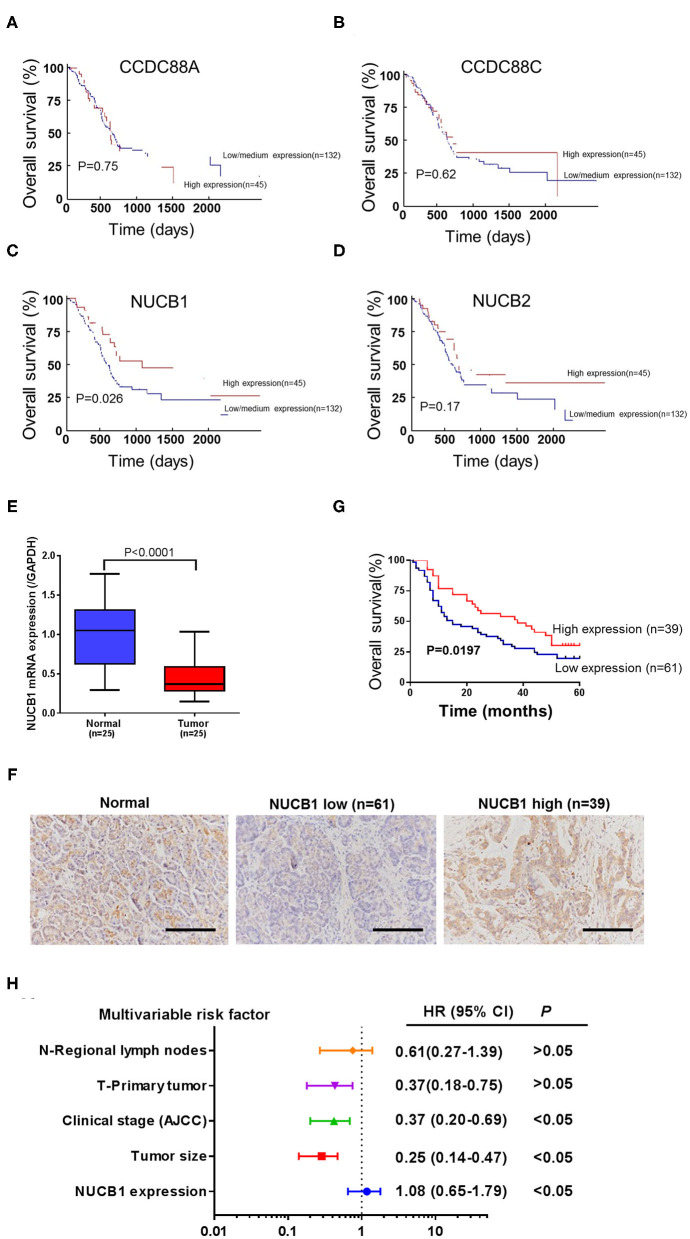
Downregulation of NUCB1 was associated with poor prognosis in PDAC patients. **(A–D)** The association between gene expression patterns and survival rates of patients with PDAC was analyzed using the UALCAN database (annotated as PAAD dataset) (http://ualcan.path.uab.edu/analysis.html). Four members of the GEF family (CCDC88A, CCDC88C, NUCB1, and NUCB2) were examined. **(E)** Quantitative PCR was used to detect NUCB1 expression in 25 pairs of pancreatic cancer and adjacent tissues. **(F)** Immunohistochemistry (IHC) was used to examine NUCB1 protein expression in tissues. *Scale bar*: 100 μm. **(G,H)** Patients were divided into two groups based on IHC staining for survival curve analysis **(G)** and multivariate regression analysis **(H)**.

Gene expression patterns and prognostic values for PDAC were analyzed from the UALCAN database (annotated as PAAD). Four members of the non-receptor GEF family, including CCDC88A, CCDC88C, NUCB1, and NUCB2, were examined (Figures 1A–D). While CCDC88A, CCDC88C, and NUCB2 expression did not correlate with prognosis in PAAD patients (Figures 1A–C), low expression of NUCB1 was highly associated with lower patient survival rate (Figure 1C). Moreover, analysis of human patient samples from Fudan University Shanghai Cancer Center (Shanghai, China) indicated that NUCB1 mRNA expression was reduced in PDAC tissues compared with corresponding adjacent tissues (*n* = 25 pairs) (Figure 1E), suggesting that NUCB1 downregulation may promote pancreatic cancer progression. We next investigated the relationship between NUCB1 expression based on immunohistochemistry (high vs. low NUCB1 expression) and overall patient survival rate in the PAAD dataset (Figure 1F). As shown in Figure 1G, high NUCB1 expression correlated with higher patient survival rate whereas low NUCB1 expression was associated with low patient survival rate. We also performed multivariate regression analysis of prognostic factors and found NUCB1 expression and clinical AJCC stage as independent prognostic factors in multivariate analysis (Figure 1H and Table 1). Moreover, the mRNA and protein expression of NUCB1 correlated (Supplementary Figure 1). Together, these data demonstrate that NUCB1 is a prognostic factor in PDAC.

**Table 1:** Correlations between NUCB1 expression and clinicopathologic features in patients with pancreatic ductal adenocarcinoma.

		**NUCB1 expression**		
**Clinicopathologic features**	**Total**	**Low (*n* = 61)**	**High (*n* = 39)**	***P* value**
**Age (years)**				
≤ 65	64	36	28	0.2089
> 65	36	25	11	
**Gender**				
Male	56	33	23	0.6830
Female	44	28	16	
**Tumor size**				
>4 cm	51	38	13	0.0074^**^
≤ 4 cm	49	23	26	
**Tumor location**				
Head	53	35	18	0.3088
Body/tail	47	26	21	
**Clinical stage (AJCC)**				
III–IV	31	24	7	
I–II	69	37	32	0.0279^*^
**T-Primary tumor**				
T3–T4	44	33	11	
T1–T2	56	28	28	0.0135^*^
**N-Regional lymph nodes**				
N1	55	41	14	
N0	45	20	25	0.0037^**^
**M-Distant metastasis**				
M0	78	47	31	0.8102
M1	22	14	8	

* *P < 0.05*,

** *P < 0.01*.

### NUCB1 Overexpression Suppressed Pancreatic Cancer Cell Proliferation and Showed Additive Effects With Gemcitabine

**Figure 2:**
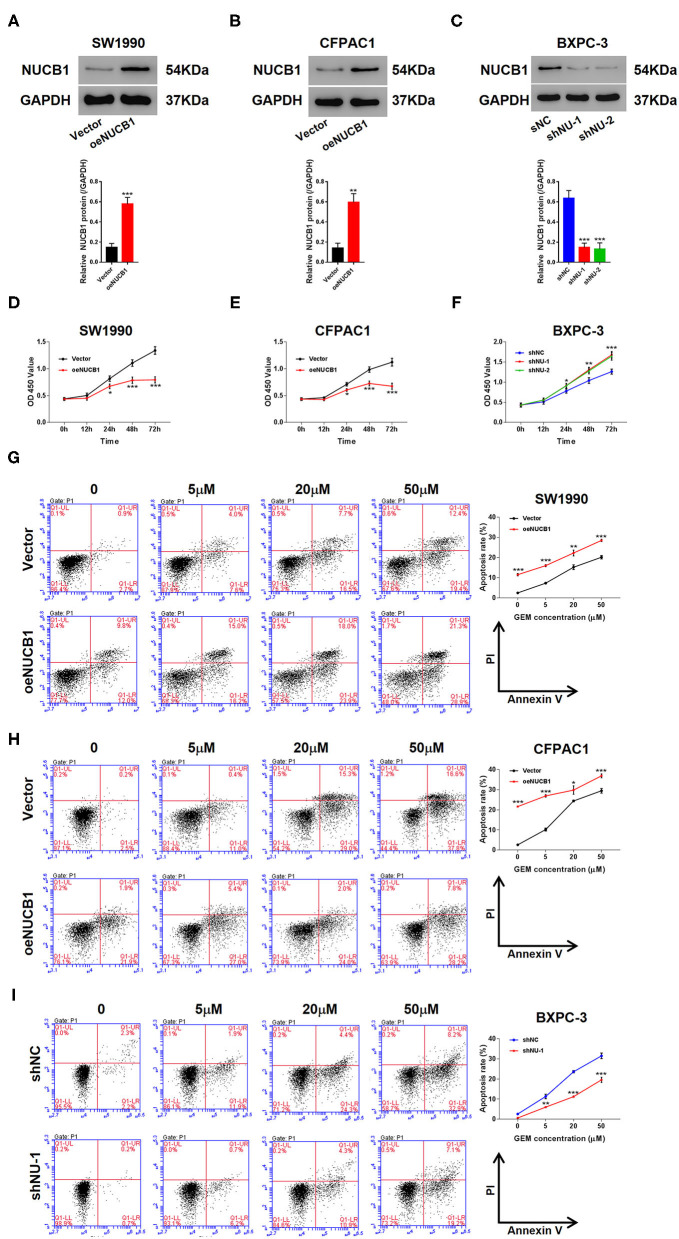
NUCB1 overexpression suppressed cell proliferation and increased the anti-tumor effects of GEM. **(A–C)** SW1990 **(A)** and CFPAC1 cells **(B)** were infected with virus expressing NUCB1 (oeNUCB1) or control vector (oeNC), and BXPC-3 cells **(C)** were infected with shRNAs targeting NUCB1 (shNU-1, shNU-2) or control shRNA (shNC). Western blot was conducted to determine protein levels. **(D–F)** CCK-8 assay was performed to assess proliferation**. (G–I)** SW1990, CFPAC1 and BXPC-3 cells were treated with 0, 5, 20, and 50 μM GEM for 24 h (0 μM represents cells treated with vehicle, DMSO) and apoptosis was detected. ^*^p < 0.05, ^**^p < 0.01, ^***^p < 0.001 vs. control (shNC or vector).

We next determined whether NUCB1-affected pancreatic cancer cell proliferation. We used two pancreatic cancer cell lines, SW1990 and CFPAC1, which relatively express lower levels of NUCB1 compared to HPDE, a normal pancreatic duct epithelial cell line (Supplementary Figure 2A), to generate stable cells with overexpression of NUCB1 or control vector (Supplementary Figures 2A,B). In parallel, we also used another cancer cell line, BXPC-3, which relatively expresses higher levels of NUCB1, to generate stable cells with NUCB1 knockdown (Supplementary Figure 2C). As shown in Figures 2A–C, overexpression or knockdown of NUCB1 correspondingly altered NUCB1 protein levels, indicating efficiency of overexpression and knockdown. Interestingly, NUCB1 overexpression in SW1990 and CFPAC1 cells significantly halted cell proliferation (Figures 2D,E), while NUCB1 knockdown dramatically increased proliferation (Figure 2F), as measured using CCK-8 assay. Furthermore, overexpression of NUCB1 in SW1990 and CFPAC1 notably increased the anti-tumor effects of gemcitabine (GEM) compared to control vector, as indicated by increased apoptosis of NUCB1-overexpressing cells compared to control cells (Figures 2G,H). Consistently, NUCB1 knockdown in BXPC-3 cells diminished the anti-tumor effects of, as indicated by reduced apoptosis in response to GEM (Figure 2I). Together, these data demonstrate important roles of NUCB1 in suppressing proliferation and enhancing the anti-tumor effects of GEM inpancreatic cancer cells *in vitro*.

### NUCB1 Overexpression Enhanced the Anti-tumor Effects of Gemcitabine *in vivo*

**Figure 3:**
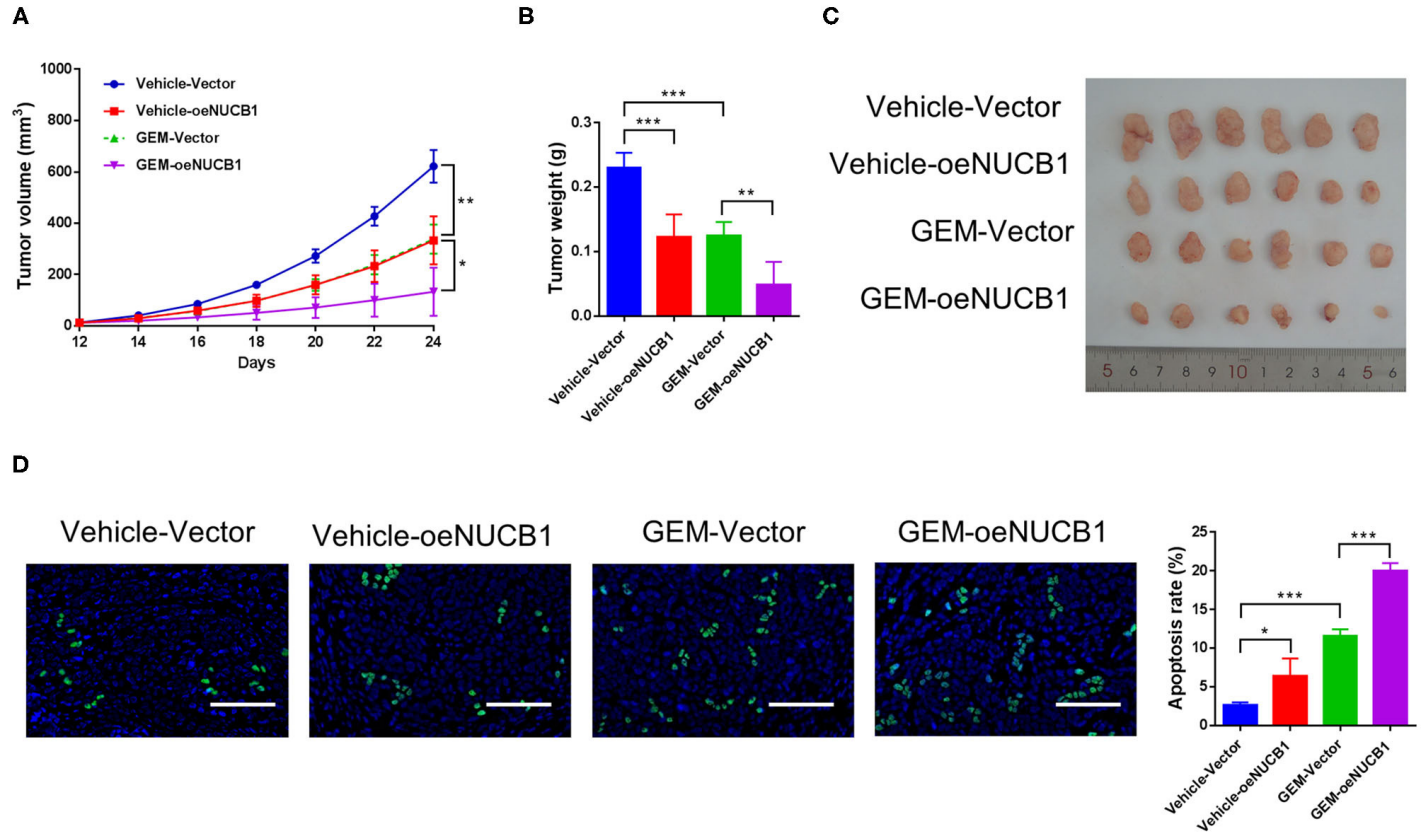
NUCB1 overexpression enhanced the anti-tumor effects of GEM *in vivo*. SW1990 cells with NUCB1 overexpression or control vector were subcutaneously injected into nude mice (*n* = 5 per group), and GEM (50 mg/kg) or vehicle was injected intraperitoneally. Xenograft growth curves **(A)** and tumor weights **(B,C)** were determined after 24 days. TUNEL staining **(D)** was conducted to detect apoptosis in xenografts. *Scale bar:* 50 μm. ^*^p < 0.05, ^***^p < 0.01, ^***^p < 0.001.

To test the additive effects of NUCB1 with GEM *in vivo*, SW1990 cells overexpressing NUCB1 or control vector were injected subcutaneously into nude mice (*n* = 6 mice per group) and GEM was injected intraperitoneally (50 mg/kg). Tumor volume was monitored and measured for 24 days. As shown in Figure 3, tumor grafts formed from SW1990 cells overexpressing NUCB1 showed additive effects with GEM treatment, as indicated by decreased tumor volumes (Figure 3A) and decreased tumor weights (Figures 3B,C). Moreover, TUNEL staining confirmed induction of apoptosis upon NUCB1 overexpression, and GEM treatment resulted in further increase in apoptosis (Figure 3D). Collectively, these data reinforce that NUCB1 suppresses proliferation and enhances the anti-tumor effects of GEM in pancreatic cancer cells *in vivo*.

### NUCB1 Suppressed GEM-Induced UPR and Autophagy

**Figure 4:**
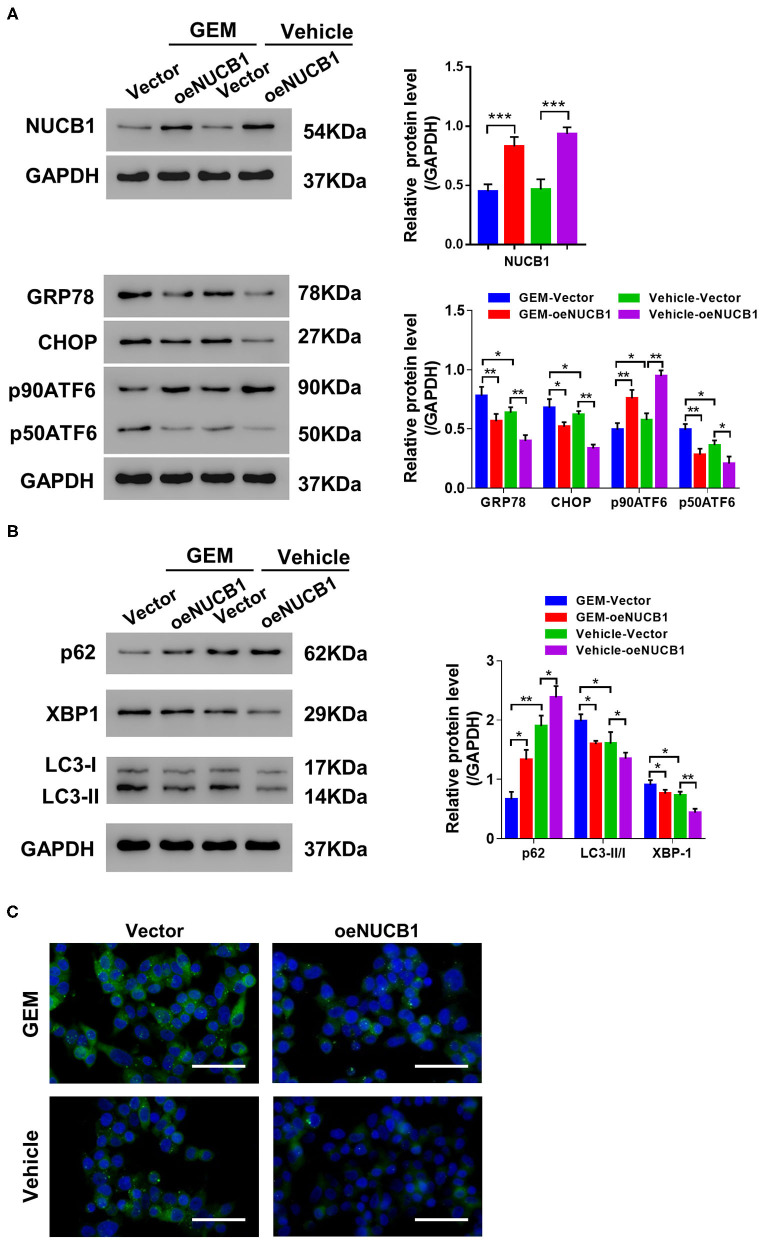
NUCB1 suppressed GEM-induced UPR and autophagy. SW1990 cells overexpressing NUCB1 were treated with 20 μM GEM. **(A,B)** Western blot was conducted to determine the proteins levels of NUCB1, UPR-associated genes (GRP78, CHOP, p90ATF6, p50ATF6) and autophagy-associated genes (p62, LC3-II/-I, and XBP1). GAPDH was used as a loading control. **(C)** Immunofluorescence staining was performed to detect LC3 expression. *Scale bar:* 50 μm. ^*^p < 0.05, ^***^p < 0.01, ^***^p < 0.001.

GEM treatment is known to induce ER stress and the UPR through multiple mechanisms, including autophagy (Wang et al., 2017). Of note, NUCB1 has been shown to control the UPR (Tsukumo et al., 2007). To determine whether the additive effects of NUCB1 with GEM in pancreatic cancer cells were linked to regulation of the UPR and autophagy, SW1990 cells overexpressing NUCB1 or control vector were treated with GEM (Figure 4A), and changes in UPR- and autophagy-associated genes were examined by Western blot. As shown in Figure 4A, GEM treatment induced the UPR, as indicated by elevated protein levels of GRP78, CHOP, and P50ATF6. Remarkably, the effects of GEM on the UPR were suppressed upon overexpression of NUCB1 (Figure 4B), indicating that NUCB1 is a critical downstream mediator of GEM-induced UPR. We also evaluated the effects of NUCB1 overexpression on the autophagosome cargo protein p62, the ER stress protein XBP1, and the autophagosome marker LC3. As shown in Figure 4B, NUCB1 overexpression antagonized the changes in the protein levels of p62 and XBP1 and the ratio of LC3-II/I induced by GEM treatment. Furthermore, NUCB1 overexpression blocked GEM-induced autophagy, as indicated by reduced immunofluorescence staining of LC3 (Figure 4C). Together, these data provide evidence that NUCB1 mediated the effects of GEM on the UPR and autophagy.

### ATF6 Reversed the Effects of NUCB1

**Figure 5:**
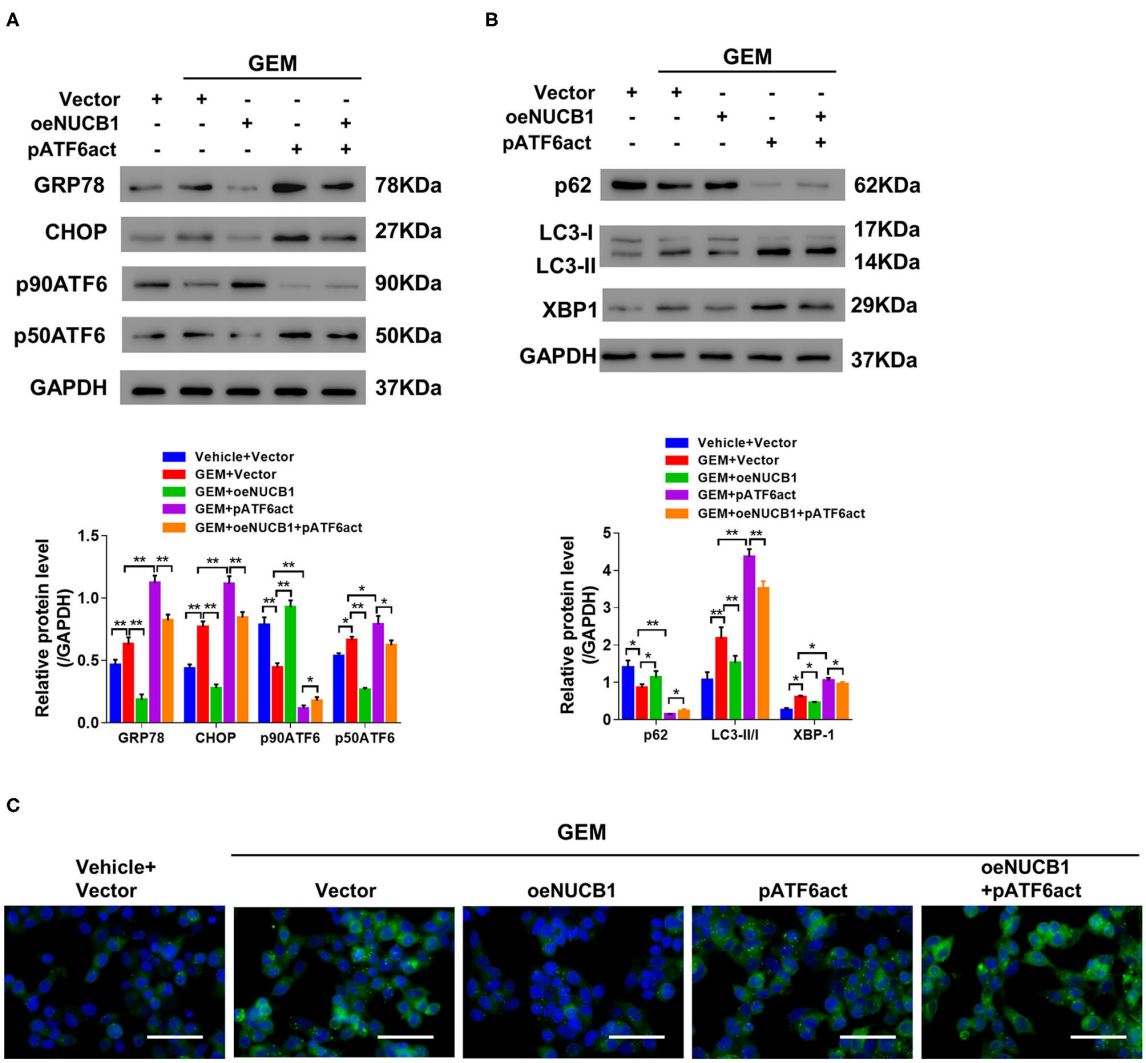
ATF6 reversed the effects of NUCB1. SW1990 cells simultaneously overexpressing ATF6-pATF6 (active) and NUCB1 were treated with 20 μM GEM. Western blot **(A,B)** was conducted to determine protein levels of GRP78, CHOP, p90ATF6, p50ATF (active), NUCB1, p62, LC3-II/-I, and XBP-1. Immunofluorescence staining **(C)** was performed to detect LC3 expression. *Scale bar:* 50 μm. ^*^p < 0.05, ^**^p < 0.01.

It has been previously reported that NUCB1 inhibits activation of ATF6 to control the UPR (Tsukumo et al., 2007). To probe this regulation in PDAC, NUCB1, and active ATF6 (pATF6act) were simultaneously overexpressed in SW1990 cells, and UPR-associated genes were analyzed. As indicated by Western blot, NUCB1 overexpression resulted in decreased protein levels of GRP78, CHOP and p50ATF6, which were rescued upon overexpression of pATF6act (Figure 5A). Furthermore, pATF6act reversed the effects of NUCB1 on autophagy, as indicated by changes in the protein level of p62, the ratio of LC3-II/I (Figure 5B) and immunofluorescence staining of LC3 (Figure 5C). Taken together, these data demonstrate that active ATF6 blocked NUCB1-mediated effects on the UPR and autophagy.

### METTL3 Regulates m^6^A Modification of NUCB1 via the m^6^A Reader YTHDF2

**Figure 6:**
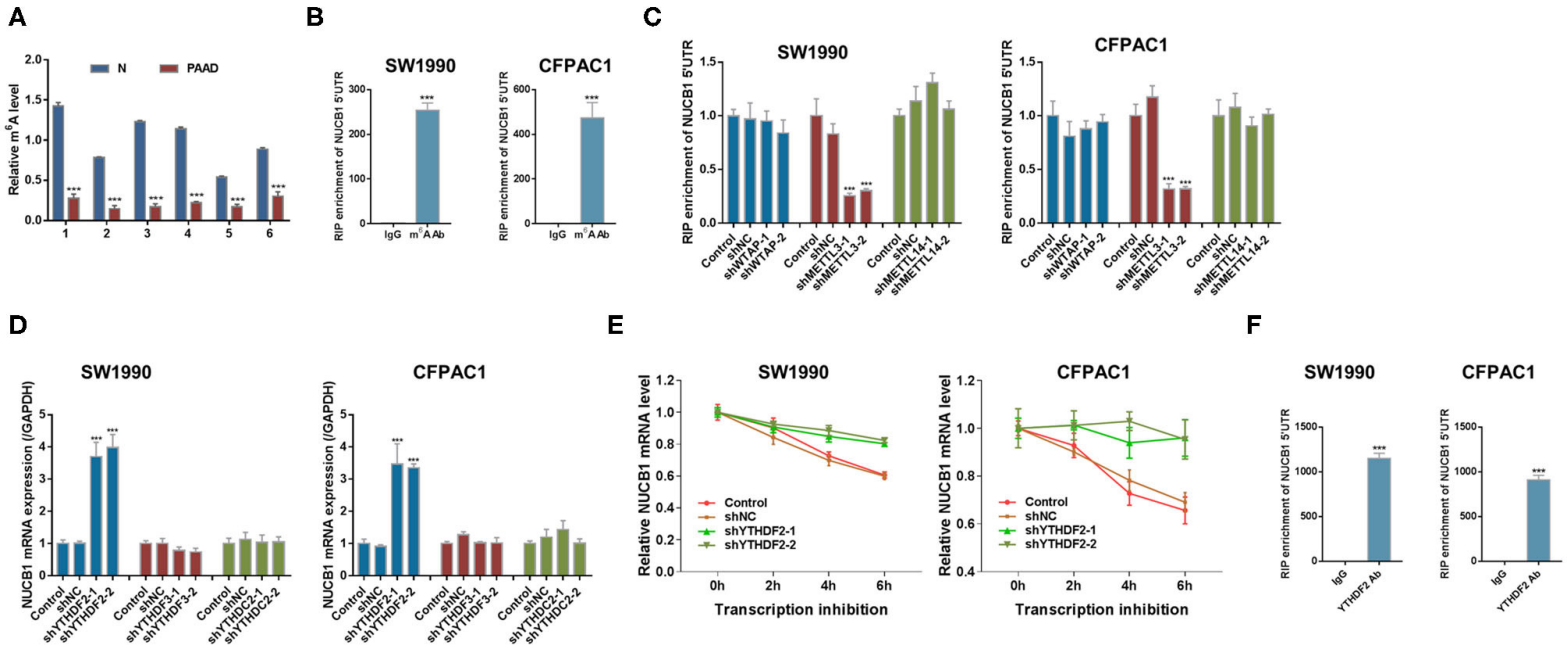
METTL3 regulates m^6^A modification of NUCB1 via the m^6^A reader YTHDF2. **(A)** m^6^A levels in six pairs of PDAC samples and adjacent non-tumorous (N) tissues were measured. ^***^p < 0.001 compared with N. **(B)** Relative m^6^A levels of NUCB1 5′-UTR in SW1990 and CFPAC1 cells were detected by RIP followed by qRT-PCR. ^***^p < 0.001 compared with IgG. **(C)** NUCB1 5′-UTR enrichment in SW1990 and CFPAC1 cells transfected with siRNAs targeting WTAP, METTL3, or METTL14 was determined by RIP followed by qRT-PCR. **(D,E)** YTHDF2 silencing in SW1990 and CFPAC1 cells increased NUCB1 mRNA levels **(D)** and stability **(E)**. ^***^p < 0.001 compared with siNC. **(F)** RIP followed by qRT-PCR was performed to examine the interaction between YTHDF2 and 5′-UTR of NUCB1 mRNA. ^***^p < 0.001 compared with IgG.

mRNA modifications have been shown to be an important factor in the post-transcriptional regulation of gene expression. m^6^A modification in mRNAs has recently emerged as a modification that plays an important role in cancer progression(Liu et al., 2020). Interestingly, we found that m^6^A levels were significantly reduced in PDAC tissues compared with adjacent non-tumorous tissues (*n* = 6 pairs, samples from Fudan University Shanghai Cancer Center) (Figure 6A). Based on our analysis (http://cuilab.cn/sramp), m^6^A methylation is predicted on NUCB1 5′UTR. To examine the interaction between NUCB1 5′UTR and m^6^A, RNA immunoprecipitation (RIP) was conducted followed by quantitative RT-PCR. As shown in Figure 6B, enrichment of NUCB1 5′UTR was detected in both SW1990 and CFPAC1 cells. m^6^A modification is emplaced by writers (Fazi and Fatica, 2019). Based on our analysis (http://m6a2target.canceromics.org), it is predicted that three writers, WTAP, METTL3, or METTL14, might regulate m^6^A modification of NUCB1. To test these predictions, we transfected SW1990 and CFPAC1 cells with shRNAs targeting WTAP, METTL13, and METTL14 (Supplementary Figures 3A–F). Strikingly, knockdown of METTL13 dramatically reduced NUCB1 5′-UTR enrichment by RIP in both SW1990 and CFPAC1 cells, whereas knockdown of WTAP and METTL14 did not affect NUCB1 5′UTR enrichment (Figure 6C). m^6^A reader proteins are able to interpret m^6^A modifications and determine the fate of the modified RNA (Fazi and Fatica, 2019). Subsequently, we tried to determine the reader of the modification in NUCB1 5′ UTR and transfected SW1990 and CFPA1 cells with shRNAs targeting YTHDF2, YTHDF3 and YTHDC2 (Supplementary Figures 4A–F). As shown in Figure 6D, knockdown of YTHDF2 significantly increased NUCB1 mRNA expression, while YTHDF3 and YTHDC2 had no obvious effects. Consistent with this, NUCB1 mRNA stability was enhanced when YTHDF2 was knocked down (Figure 6E). Furthermore, YTHDF2 interacted with 5′UTR of NUCB1 mRNA by RIP and qRT-PCR (Figure 6F). Collectively, these results demonstrate that METTL3 promotes m^6^A modification of NUCB1 5′UTR via the m^6^A reader YTHDF2.

## Discussion

**Figure 7:**
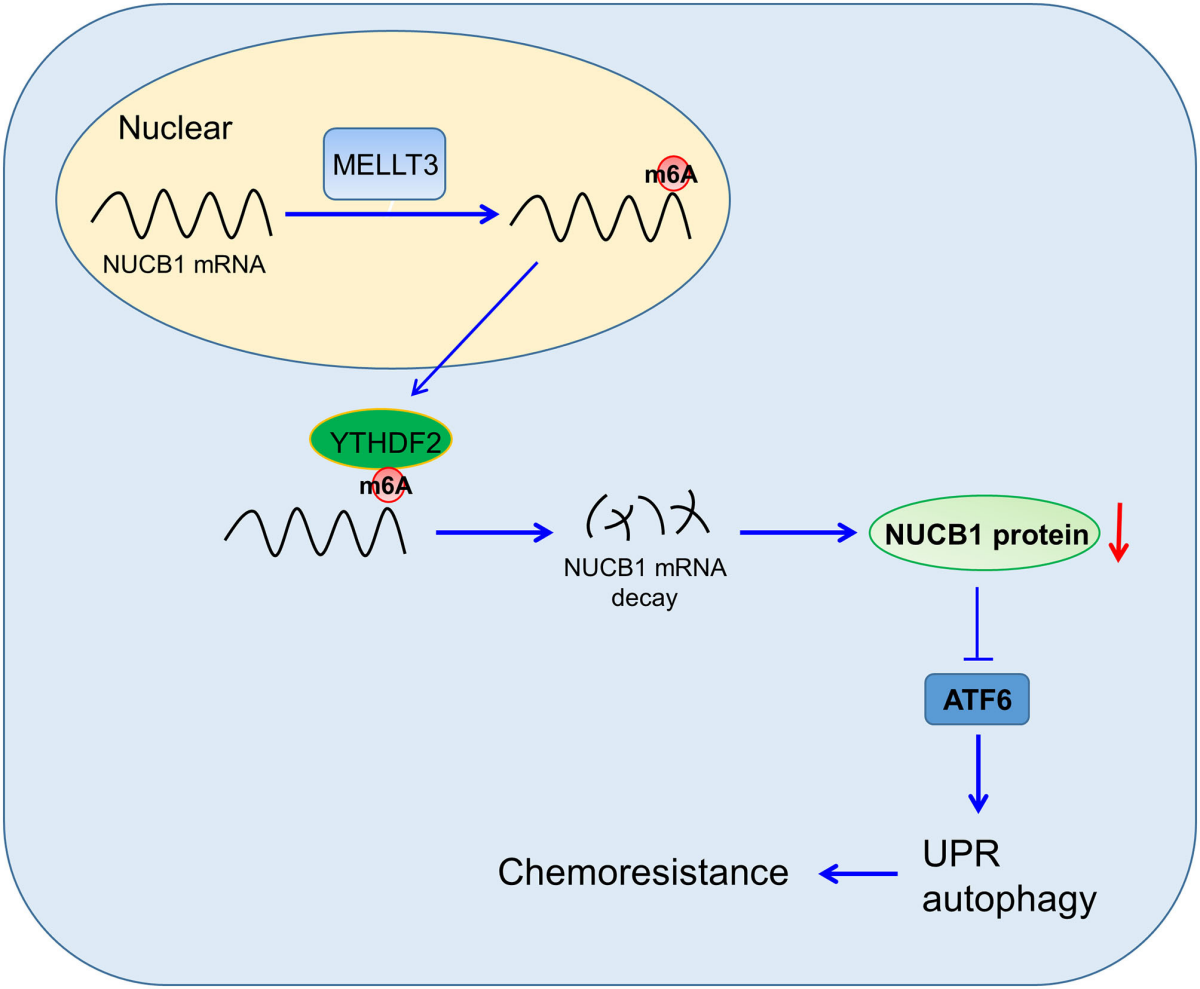
Proposed Model of Regulation. NUCB1 regulates ATF activity to modulate UPR and autophagy. METTL3 promotes m^6^A modification of NUCB1 via YTHDF2.

PDAC is a highly aggressive cancer, and understanding the mechanisms that regulate disease progression is key to developing successful therapeutics. In this study, we provided evidence for a role of NUCB1 in regulating proliferation and the anti-tumor effects of gemcitabine in pancreatic cancer cells, and proposed a mechanism whereby METTL3 controls NUCB1 expression, which then modulates ATF activity and subsequently controls the UPR (Figure 7).

Consistent with the results from a previous study (Tsukumo et al., 2007), our findings support that NUCB1 functions to inhibit ATF6 activation, establishing an important molecular link between NUCB1 and ER stress/UPR. It is worth noting that a previous report that investigated the functions of non-receptor GEFs in metastatic colorectal cancer found that NUCB1 overexpression was correlated with shorter progression-free survival (PFS) (Barbazan et al., 2016). Our study reveals for the first time the function of NUCB1 in PDAC and suggests a tumor-suppressive role. Thus, NUCB1 may play context-dependent roles in cancer.

One important clinical implication of our findings relates to the role of NUCB1 in enhancing the anti-tumor effects of gemcitabine, a first-line chemotherapy for pancreatic cancer patients (Heinemann, 2001). Drug resistance is an enormous challenge in PDAC treatment and is one of the key factors responsible for therapeutic failures in PDAC. The additive effects of NUCB1 with gemcitabine in pancreatic cancer cells suggest that targeting NUCB1, in combination with other current treatment strategies, may improve therapeutic efficacy and clinical outcomes for PDAC patients. Further functional analysis with primary tumor cells will strength our current findings in the future.

The downregulation of NUCB1 expression by METTL3 represents another important layer of regulation that can be exploited to develop treatment strategies for PDAC. Evidence suggests that m^6^A methylation may contribute to PDAC progression (Chen et al., 2019). METTL3 is overexpressed in PDAC and has been shown to contribute to chemoresistance of pancreatic cancer cells (Taketo et al., 2018). In support of this, METTL3 knockout in pancreatic cancer cells increased sensitivity to gemcitabine, 5′-fluorouracil and cisplatin (Taketo et al., 2018). Similarly, overexpression of the m^6^A reader YTHDF2 has been reported in pancreatic cancer, particularly in patients at later stages of cancer progression (Chen et al., 2017; Pinello et al., 2018). Consistently, inhibition of YTHDF2 expression in pancreatic cancer cells suppresses proliferation and colony formation (Chen et al., 2017), highlighting an oncogenic role for YTHDF2 in PDAC. We so found that YTHDF2 knockdown elevated gemcitabine-induced apoptosis in SW1990 cells (Supplementary Figure 5). Together, our findings and those of others suggest that m^6^A modification may be a promising target for PDAC treatment.

Finally, how NUCB1 contributes to PDAC cancer progression, as well as the mechanism through which it influences the anti-tumor effects of gemcitabine in pancreatic cancer cells, have yet to be elucidated. It will be interesting to see whether NUCB1 affects DNA repair as this has been suggested to influence drug response (Quinonero et al., 2019). Understanding the functions of NUCB1 during homeostasis is also crucial in gauging the global effects of perturbing its expression in PDAC. Whether there are additional downstream targets and mediators of NUCB1 in PDAC and, if so, their precise contribution, also need to be investigated in future studies. In sum, our study uncovered a novel role for NUCB1 in PDAC progression by controlling the UPR via ATF6 activity and highlights a regulatory mechanism involving METTL3-mediated regulation of NUCB1.

## Data Availability Statement

The datasets presented in this study can be found in online repositories. The names of the repository/repositories and accession number(s) can be found in the article/Supplementary Material.

## Ethics Statement

The studies involving human participants were performed following the protocol reviewed and approved by the Institutional Ethical Review Committee of Fudan University Shanghai Cancer Center (Shanghai, China). The patients/participants provided their written informed consent to participate in this study. All the described animal experiments were approved by the Animal Care Committee of Fudan University Shanghai Cancer Center (Shanghai, China).

## Author Contributions

Y-QH and L-ML designed the experiments and wrote the manuscript. Y-QH, KZ, JS, Z-YN, YL, and W-dS performed the experiments and the statistical analysis. L-ML supervised the study. All authors have read and approved the final version of the manuscript.

## Conflict of Interest

The authors declare that the research was conducted in the absence of any commercial or financial relationships that could be construed as a potential conflict of interest.
