# Supplementary material for: NUCB1 Suppresses Growth and Shows Additive Effects With Gemcitabine in Pancreatic Ductal Adenocarcinoma via the Unfolded Protein Response
Source: Front Cell Dev Biol. 2021 Mar 29;9:641836. doi: 10.3389/fcell.2021.641836 (PMC8041069; doi:10.3389/fcell.2021.641836)
Supplement: Supplementary file 1 [file Data_Sheet_1.DOCX]

**Table S1.** Primer sequences for real-time PCR.

| **Gene** | **Forward primer** | **Reverse primer** |
| --- | --- | --- |
| NUCB1 | 5' ACGGCGTTATCTGGAGTC 3' | 5' TCCTCATTCTTTGGGTCG 3' |
| WTAP | 5' GTAATGCGACTAGCAACC 3' | 5' TATCAGGCGTAAACTTCC 3' |
| METTL3 | 5'CCAGATGCTCCTGCCACTC3' | 5'ACAGTCCCTGCTACCTCCC3 |
| METTL4 | 5' CCCATGTACTTACAAGCC 3' | 5' CAGTGATGCCAGTTTCTC 3' |
| YTHDF2 | 5'GGCAGCACTGAAGTTGGG 3' | 5'GTGGCGGAAGACTTGACC 3' |
| YTHDF3 | 5'AAAGATGTTCCCAATAACC 3' | 5'TTCCTCCTCTTCTTGACG 3' |
| YTHDC2 | 5' ATTGATGGCAGGAGATAG 3' | 5'CAAGGCAGCACTAGAAAG 3' |
| GAPDH | 5' AATCCCATCACCATCTTC 3' | 5' AGGCTGTTGTCATACTTC 3' |

**Table S2.** shRNA sequences used in this study.

| **Gene** | **shRNA** | **Target sequence** |
| --- | --- | --- |
| NUCB1 | shNU-1 | 5’ CCAATGCGGAGGACATCAA 3’ |
|  | shNU-2 | 5’ CCCATCATGAAGAGTTCAA 3’ |
|  | shNU-3 | 5’ GGGAGCATGTGATGAAGAA 3’ |
| WTAP | shWTAP-1 | 5’ GCAAGTACACAGATCTTAA 3’ |
|  | shWTAP-2 | 5’ GCGAAGTGTCGAATGCTTA 3’ |
| METTL3 | shMETTL3-1 | 5’GCTGCACTTCAGACGAATT3’ |
|  | shMETTL3-2 | 5’ GGATACCTGCAAGTATGTT 3’ |
| METTL4 | shMETTL4-1 | 5’ GCATTGGTGCCGTGTTAAA 3’ |
|  | shMETTL4-2 | 5’ GCTGACAGATTTGAAGAAT 3’ |
| YTHDF2 | shYTHDF2-1 | 5’ GCACAGAAGTTGCAAGCAA 3’ |
|  | shYTHDF2-2 | 5’ TTGCTTGCAACTTCTGTGT 3’ |
| YTHDF3 | shYTHDF3-1 | 5’ CCTATGGACAAATGAGTAA 3’ |
|  | shYTHDF3-2 | 5’ GCAGTGGTATGACTAGCAT 3’ |
| YTHDC2 | shYTHDC2-1 | 5’ GCGACTCAACAATGGCATA 3’ |
|  | shYTHDC2-2 | 5’ GGAAATGGATGCTTGCCTT 3’ |

**Table S3.** Antibody list.

| **Primary antibody** | **Company** | **Catalog No.** |
| --- | --- | --- |
| NUCB1 | Abcam | Ab154262 |
| GRP78 | Abcam | Ab108615 |
| P50ATF6(active) | Bioss | bs-1634R |
| P90ATF6 | Bioss | bs-1634R |
| P62 | Abcam | Ab109012 |
| CHOP | Cell Signaling Technology | #2895 |
| XBP1 | Abcam | Ab37152 |
| LC3 | Abcam | Ab48394 |
| WTAP | Abcam | Ab195380 |
| METTL3 | Abcam | Ab195352 |
| METTL4 | Abcam | Ab220030 |
| YTHDF2 | Abcam | Ab220163 |
| YTHDF3 | Abcam | Ab220161 |
| YTHDC2 | Abcam | Ab220160 |
| GAPDH | Cell Signaling Technology | #5174 |


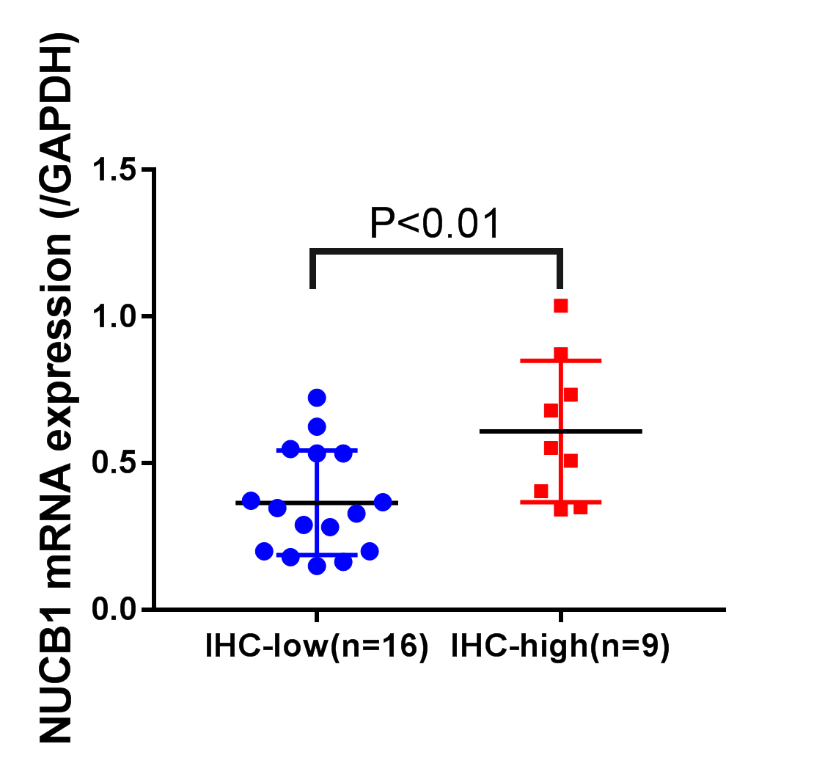


**Figure S1.** mRNA and protein expression of NUCB1 in PDAC showed a positive correlation. PDAC samples in Figure 1E were also subjected to immunohistochemical (IHC) staining, and divided into immunohistochemical (IHC)-low expression and –high expression group.


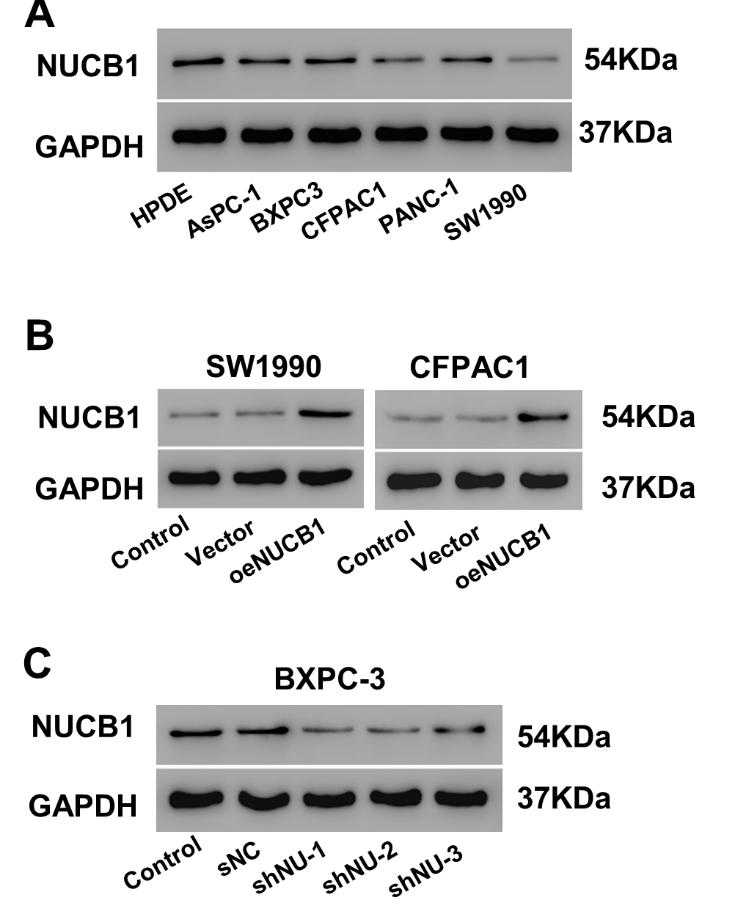


**Figure S2.** **A.** NUCB1 protein expression in PAAD lines. **B.** SW1990 and CFPAC1 cells were infected with lentivirus overexpressing NUCB1 (oeNUCB1) or control vector (oeNC). **C.** BXPC-3 cells were infected with lentivirus expressing NUCB1 shRNAs (shNU-1 and shNU-2) or control shRNA (shNC). NUCB1 protein levels were determined by Western blot.


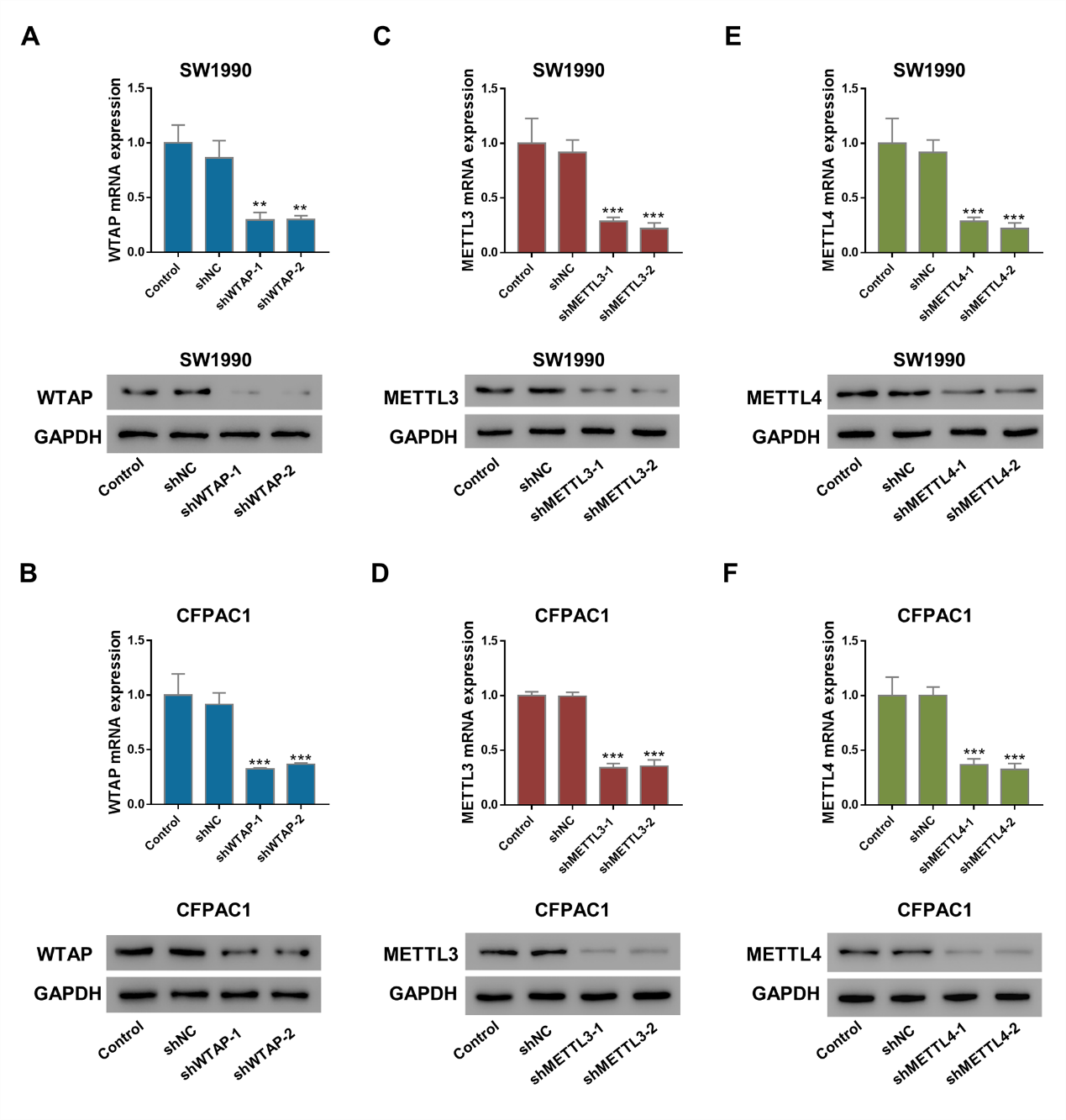


**Figure S3. A-E.** SW1990 and CFPAC1 cells were infected with lentivirus expressing shRNAs for WTAP, METTL3 or METTL4. mRNA expression and protein levels were detected by qRT-PCR (A, C, E) and Western blot (B, D, F), respectively. ****p*<0.001 vs shNC.


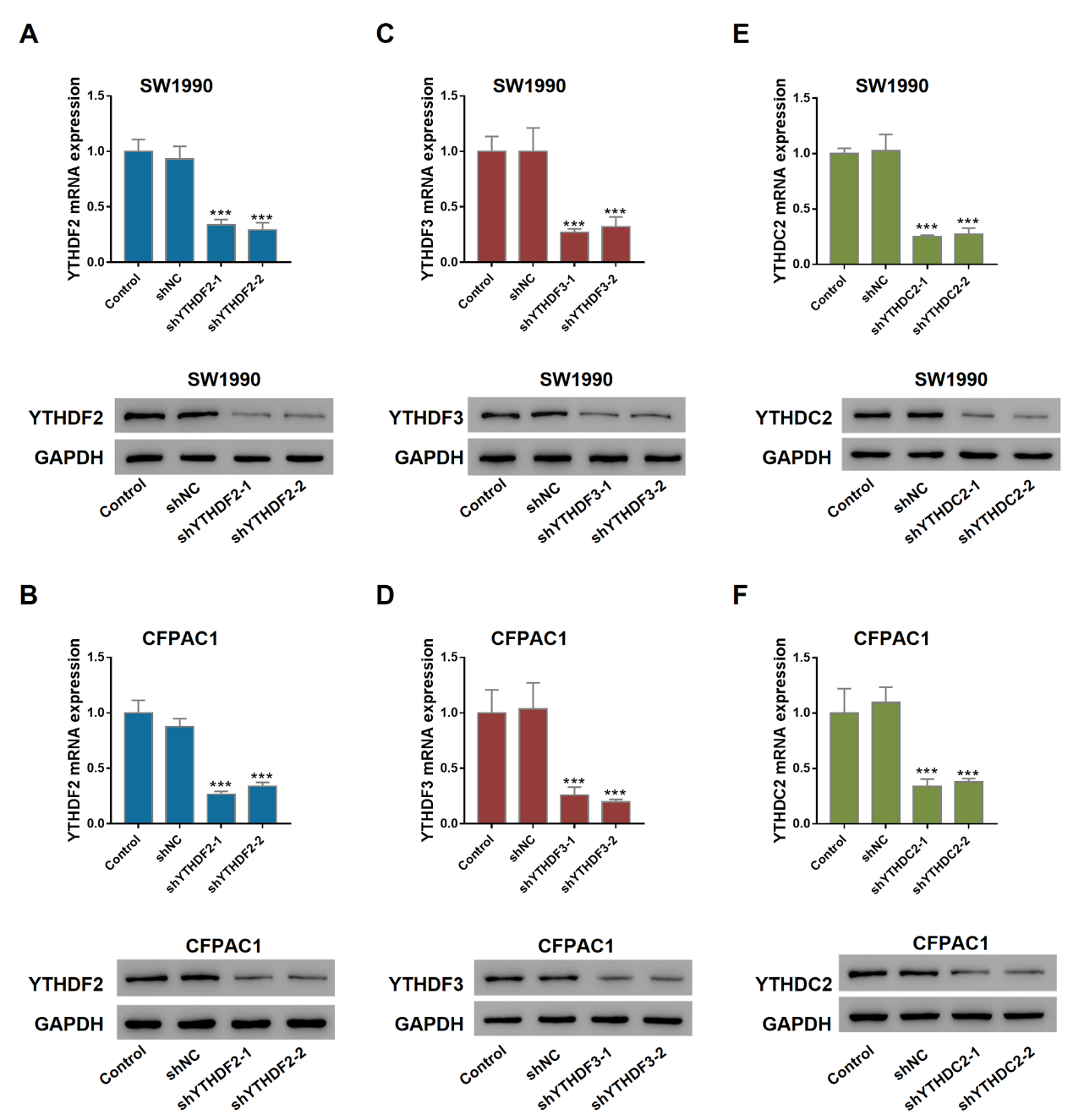


**Figure S4. A-E.** SW1990 and CFPAC1 cells were infected with lentivirus expressing shRNAs for YTHDF2, YTHDF3 or YTHDC2. mRNA expression and protein levels were determined by qRT-PCR and Western blot, respectively. ****p*<0.001 vs shNC.


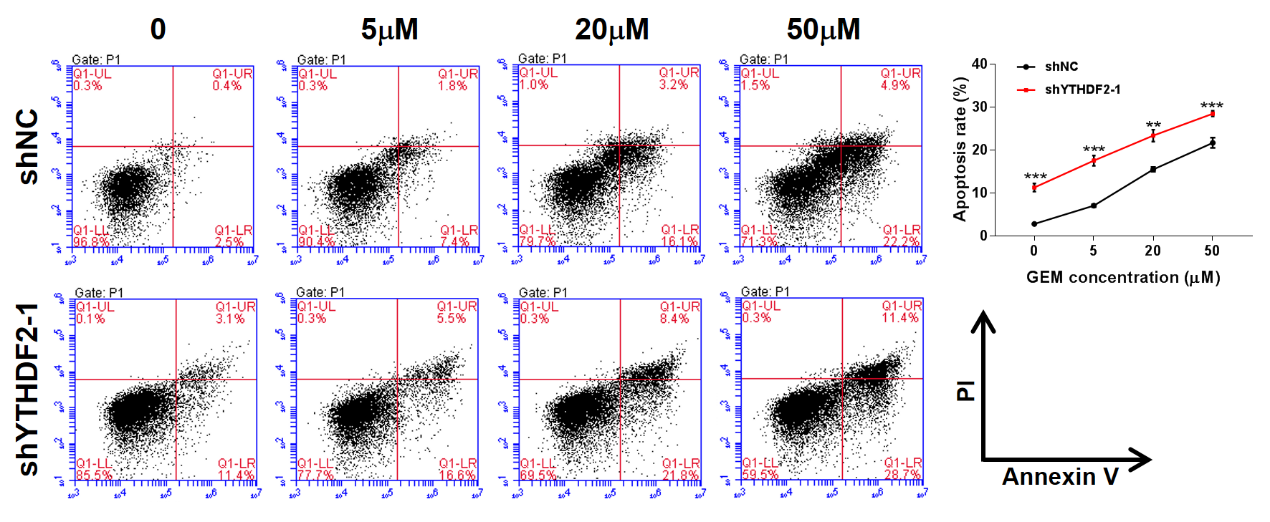


**Figure S5.** SW1990 cells with YTHDF2 knockdown were treated with 0, 5, 20 and 50 μM GEM for 24 hours (0 μM represents cells treated with vehicle, DMSO) and apoptosis was detected. **p<0.01, ****p*<0.001 vs shNC.
